# Therapeutic and preventive strategies based on the maladaptive plasticity hypothesis for Alzheimer’s disease

**DOI:** 10.3389/fnagi.2025.1726144

**Published:** 2026-01-20

**Authors:** Shigeki Kawabata

**Affiliations:** Sompo Holdings, Inc., Tokyo, Japan

**Keywords:** activity-based intervention, Alzheimer’s disease, amyloid-β precursor protein, early endosome, membrane trafficking, network abnormality, synaptic remodeling, synaptic vesicle cycling

## Abstract

Alzheimer’s disease (AD), the most common form of dementia, is characterized by two hallmark pathologies, amyloid plaques (APs) and neurofibrillary tangles (NFTs). Amyloid-β and tau, key components of APs and NFTs, respectively, are widely considered primary drivers of neurodegeneration in AD. In contrast, an alternative view proposes that network failure, arising from amyloid-*β* precursor protein-driven excessive/aberrant and maladaptive synaptic plasticity, underlies AD pathophysiology. Synaptic plasticity is indispensable for cognitive functions such as learning and memory; however, when dysregulated, it may lead to cognitive decline and accelerate the trajectory toward AD. This paper, based on this hypothesis, examines strategies to mitigate maladaptive plasticity while preserving adaptive plasticity, and proposes the potential of novel approaches for the prevention and treatment of mild cognitive impairment and AD, encompassing both activity-based interventions and pharmacological treatments. This hypothesis-driven framework offers a coherent perspective linking molecular, circuit, and cognitive levels of dysfunction in AD, and may guide more integrative, multi-level approaches to future preventive and therapeutic strategies, a direction increasingly emphasized in current experimental and clinical AD research.

## 1 Introduction

**Figure 1:**
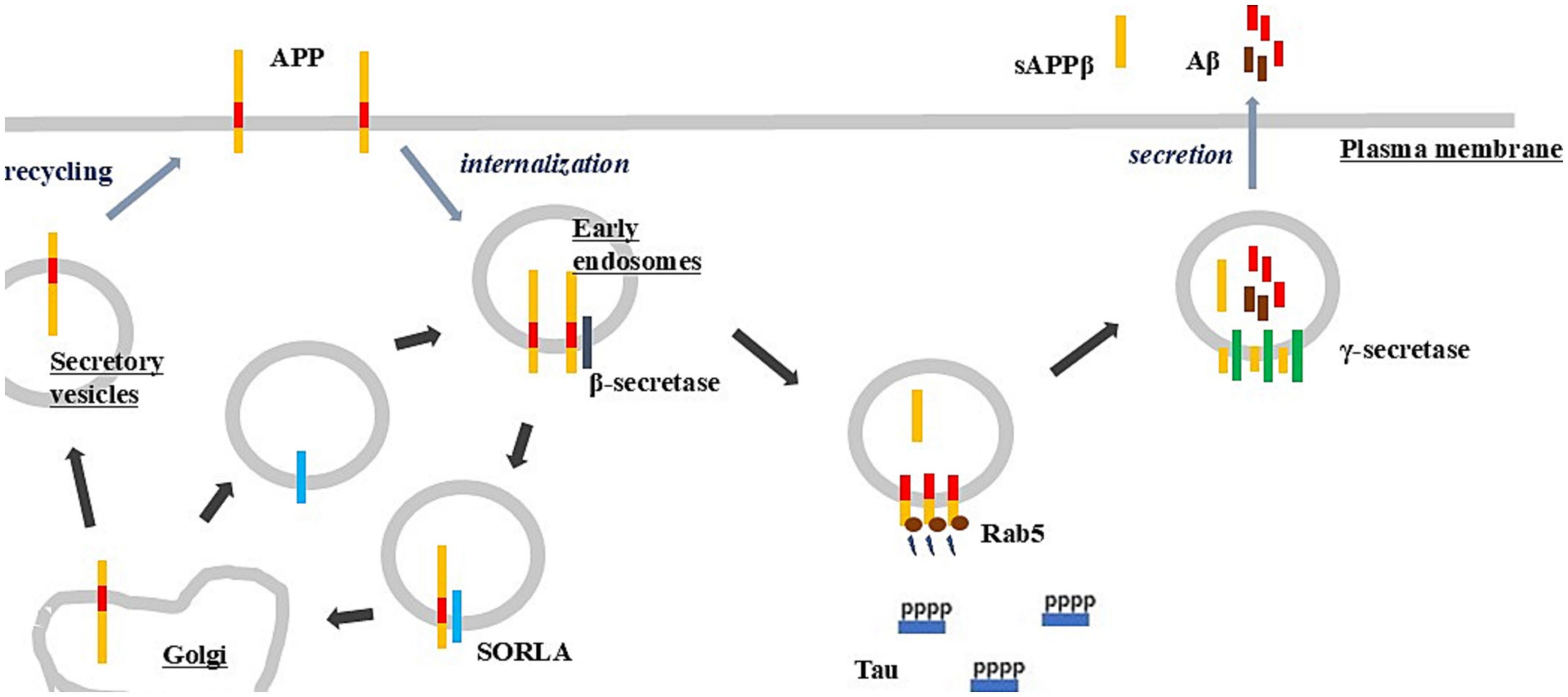
Intracellular trafficking and signaling of the amyloid-β precursor protein (APP). APP is transported through the secretory and endocytic pathways. Following endocytosis, β-secretase cleavage generates CTF-β within early endosomes, and subsequent γ-secretase processing produces Aβ. Activation of Rab5 by CTF-β may contribute to tau phosphorylation, whereas SORLA promotes the recycling of APP back to the plasma membrane.

The amyloid-*β* precursor protein (APP) gene is causative for familial Alzheimer’s disease (FAD). APP plays a crucial role in neuronal development, including axonal formation and synaptogenesis, as well as in the remodeling of synaptic architecture that underlies structural synaptic plasticity. Here, synaptic plasticity refers to the ability of neural circuits to remodel synaptic structures, supporting the formation of learning and memory and enabling adaptive responses to neuronal injury. *Drosophila* has an APP homolog, APPL. Studies on APP-like (APPL)-knockout flies demonstrate that APPL is indispensable to axonal formation and synaptogenesis in the development of the Mushroom Body (Soldano et al., 2013) and the neuromuscular junction (Torroja et al., 1999). Mammals such as mice and humans have two partially redundant APP homologues, APP-like proteins 1 and 2 (APLP1 and APLP2), and mice doubly deficient in APP and APLP2 exhibit a severe defect of neuromuscular synapse formation and early postnatal lethality (Wang et al., 2005). Studies in genetically modified mice, *Drosophila* flies, and rodent neuronal cultures demonstrate that the C-terminal fragment (CTF) of APP is indispensable to these functions (Deyts et al., 2012; Klevanski et al., 2015; Leyssen et al., 2005; Soldano et al., 2013; Torroja et al., 1999). Proteolytic processing of APP produces two types of CTF, namely CTF-α and CTF-β. Within the non-amyloidogenic α-pathway, at the cell surface, α-secretase produces secreted sAPPα and the membrane anchored CTF-α (Koo and Squazzo, 1994). In the amyloidogenic β-pathway, β-secretase (the β-site APP cleaving enzyme 1 [BACE1]) cleaves APP in early endosomes, resulting in the generation of sAPPβ and CTF-β (Winckler et al., 2018) (Figure 1). CTF-β is subsequently cleaved by *γ*-secretase, and amyloid-β (Aβ) is produced from CTF-β. The catalytic core of γ-secretase complex is presenilin (PSEN1 and PSEN2), which is also causal for FAD. In P-domain lamellipodia, APP colocalizes with Ras-related protein in brain 5 (Rab5), which may be involved in neurite outgrowth and guidance, and regulate actin-based growth cone motility (Sabo et al., 2003). CTF-β, but not CTF-*α*, activates Rab5 and induces increased active Rab5 levels in early endosomes (Kim et al., 2016; Xu et al., 2016) (Figure 1), indicating that CTF-β is a domain of APP, which is involved in synapse formation and its remodeling. In iPSC-derived neurons carrying various FAD-linked APP and/or PSEN1 mutations, CTF-β and Rab5 commonly increase in the endosomal compartment (Kwart et al., 2019). Consistent with these *in vitro* findings, *in vivo* studies have shown that during juvenile stages, APPswe/PS1dE9 mice show abnormal reorganization of hippocampal synaptic structures, whereas Tg2576 mice show increased synapse numbers in both the hippocampus and cerebral cortex. In both models, these early changes are followed by a decline in synapses with aging (Lee et al., 2010; Ray et al., 2024). In mice expressing M146V PSEN1 mutant, exuberant neurite outgrowth and hippocampal axonal sprouting are seen (Deyts et al., 2016). The early hypersynchrony and subsequent decline of the default mode network (DMN) observed in individuals carrying the E280A PSEN1 mutation (Guzmán-Vélez et al., 2022; Quiroz et al., 2015), and in AD mouse models expressing FAD-linked mutant gene(s) (Ben-Nejma et al., 2019; Bero et al., 2012; Shah et al., 2018), may be a consequence of plasticity-related functional abnormalities arising from FAD-associated mutations in APP and presenilin. Similar patterns of early DMN hypersynchrony and later decline have also been observed in Apolipoprotein E (APOE) ε4 carriers (Badhwar et al., 2017; Filippini et al., 2009; Koelewijn et al., 2019; Su et al., 2015).

In mild cognitive impairment (MCI) patients, hypoactivity of the DMN is evident in the precuneus and posterior cingulate cortex (PCC) (Bai et al., 2008; Qi et al., 2010; Talwar et al., 2021; Wang et al., 2018). Conversely, increased DMN activity has been observed in other hub regions, such as the medial frontal lobe (MFL) and inferior parietal lobule (Bai et al., 2008; Qi et al., 2010; Wang et al., 2018). In the medial temporal lobe (MTL), neuronal hyperactivation during task performance is observed in individuals with early MCI (Corriveau-Lecavalier et al., 2021b; Zott et al., 2018). Such network hyperactivities are often interpreted as evidence of adaptive compensatory plasticity (successful compensatory response). However, compensatory responses are not always successful; when dysregulated, they can ultimately give rise to maladaptive neuronal connections (Grady, 2012). In AD, levels of the growth-associated protein, GAP-43 are increased in the cerebrospinal fluid (CSF) and higher levels are associated with poorer cognitive performance and faster clinical deterioration (Öhrfelt et al., 2023; Qiang et al., 2022). Longitudinally, progressive MCI patients show increasing connectivity in the anterior part of the DMN compared with stable MCI patients, and the rate of connectivity change is significantly associated with the rate of cognitive decline (Malotaux et al., 2023). In older adults with early-stage MCI or subjective cognitive impairment, greater activation of neurons in the hippocampus or MTL during the task is associated with poorer memory performance (Corriveau-Lecavalier et al., 2021a; Dickerson et al., 2004; Yassa et al., 2010) and worse prognosis, namely, faster decline of cognitive functions and higher rate of AD development (Dickerson et al., 2004; Dickerson and Sperling, 2008; Huijbers et al., 2015; Miller et al., 2008; O'Brien et al., 2010). The progressive loss of neurons and synapses with aging may increase the demand for synaptic plasticity as a compensatory mechanism (Burke and Barnes, 2006; Grady, 2012; Petralia et al., 2014). While such compensatory plasticity may initially be adaptive, increasing demands may lead to errors in the remodeling process and potentially drive the system into maladaptive forms, which may precipitate cognitive decline and drive the pathophysiological cascade of AD. In this context, maladaptive synaptic plasticity refers to excessive, aberrant, or misdirected synaptic remodeling that disrupts homeostatic regulation, impairs circuit function, and contributes to neural dysfunction.

Synaptic remodeling is tightly coupled with cytoskeletal reorganization, in which tau plays a key role through regulating microtubule assembly. During this dynamic process, phosphorylation of tau reduces its binding affinity to microtubules, thereby destabilizing microtubule bundles. Subsequent tau dephosphorylation promotes their reassembly and facilitates cytoskeletal reorganization. Burdensome works during synaptic plasticity perturb remodeling processes, leading to cytoskeletal disorganization and neurodegeneration. When synaptic remodeling occurs in an excessive/aberrant manner, maladaptive neuronal connections may form, resulting in cognitive dysfunction, as well as neuronal death due to glutamate excitotoxicity and energy failure. Consequently, initial network hyperexcitability leads to progressive neuronal impairment and cell loss, ultimately culminating in hypoactivity of the neuronal network.

In my previous work, I proposed that excessive/aberrant and maladaptive synaptic plasticity may represent a plausible and integrative pathophysiological mechanism in AD (Kawabata, 2022a,b). In this paper, I discuss the potential application of therapeutic strategies aimed at mitigating maladaptive synaptic remodeling for the prevention and treatment of MCI and AD.

## 2 APP expression, metabolism, cellular trafficking, and signaling

### 2.1 BACE1 inhibitors

Palmqvist et al. (2017) applied a novel methodology of combining data of Aβ levels in Positron Emission Tomography (PET) study and levels of Aβ42 in the CSF, and succeeded in identifying individuals who had just recently started to accumulate Aβ in the brain. This longitudinal study in healthy older adults revealed that Aβ accumulation preferentially begins in the precuneus/PCC and MFL regions of the DMN, and is accompanied by a decline in DMN activity, and thereafter hypometabolism and atrophy in these regions. Notably, it was demonstrated that PCC and MFL exhibit increased activity before the formation of APs, coinciding with the period during which CSF Aβ42 declines toward abnormal levels (Palmqvist et al., 2017). BACE1 expression is known to be upregulated in sprouting axons during the course of synaptic remodeling (Yan et al., 2012a,b). The CTF-β produced by BACE1 promotes synaptic remodeling, and subsequent *γ*-secretase cleavage of CTF-β leads to increased Aβ generation. Accordingly, the concomitant emergence of DMN hyperactivation and abnormal Aβ metabolism observed in cognitively normal older adults may suggest that an age-related increase in compensatory responses leading to elevated BACE1 activity may contribute to both DMN hyperactivation and the initiation of Aβ accumulation.

BACE1 inhibitors have been developed as a target molecule based on the amyloid hypothesis, but it may be also a candidate molecular target under the excessive/aberrant and maladaptive synaptic plasticity hypothesis (hereafter referred to as the maladaptive plasticity hypothesis). However, numerous clinical trials of BACE1 inhibitors have failed due to safety and efficacy issues (Coimbra et al., 2024; Ghosh, 2024). These adverse outcomes are attributed to the fact that BACE1 is a protease with multiple substrates. In addition, many inhibitors exhibit low selectivity against BACE2, the other isoform of BACE. Adverse effects include cognitive worsening. The seizure protein 6 (Sez6) family members (Sez6, Sez6-Like 1, and Sez6-Like 2), substrates of BACE1 (Kuhn et al., 2012) regulate dendritic spine morphology and synaptic transmission efficiency. In triple knockout mice lacking all Sez6 family members, reduced spine density in the hippocampus has been reported, along with deficits in working memory and spatial short-term memory (Nash et al., 2020), suggesting that processing of Sez6 family proteins is implicated in the cognitive side effects of BACE1 inhibitors. APP also plays an important role in synaptic remodeling, and conditional double knockout mice lacking both APP and its functionally redundant homolog APLP2 exhibit impaired synaptic plasticity and learning deficits (Hick et al., 2015; Mehr et al., 2020). APLP1 and APLP2, members of the APP protein family, are also substrates of BACE1 (Kuhn et al., 2012). Thus, BACE1 inhibitor-mediated suppression of APP family protein processing may also underlie the cognitive deficits reported as adverse effects. To overcome the adverse effects of BACE1 inhibitors, strategies such as improving selectivity over BACE2 and refining substrate specificity have been proposed (Coimbra et al., 2024; Ghosh, 2024). However, it remains essential to consider potential on-target side effects affecting APP family proteins.

Additional proteins involved in the regulation of APP metabolism have also been reported, and one such protein is signal peptide peptidase-like 2B (SPPL2b). SPPL2b promotes APP processing by facilitating the cleavage of Integral membrane protein 2B (ITM2B), also known as Bri2, an inhibitory modulator of APP metabolism, thereby enhancing the production of Aβ and sAPPβ (Maccioni et al., 2024). Thus, inhibition of SPPL2b may also represent a potential therapeutic target.

### 2.2 Suppression of gene expression

One approach to suppress APP function involves the use of antisense oligonucleotide technology (Thirumalai et al., 2024). Among these, ALN-APP has already entered clinical trials and has been shown to produce a robust and durable reduction of CSF sAPPβ without severe adverse effects.^1^ Although a more detailed assessment of side effects remains to be conducted, this approach may represent a means to avoid on-target adverse effects associated with suppression of APP family protein functions, while preserving appropriately regulated compensatory responses and preferentially suppressing maladaptive compensatory responses implicated in the pathogenesis of AD. APP is known as a central molecule in the pathogenesis of AD. According to the maladaptive plasticity hypothesis, dysregulation of APP function is thought to induce abnormal plastic changes, leading to maladaptive responses. In contrast, APLP2 shares overlapping functions with APP and is known to partially compensate for its absence in neural contexts (Hick et al., 2015; Mehr et al., 2020). Therefore, selectively targeting APP itself may represent a rational strategy to minimize adverse effects. While suppression of APP function could attenuate maladaptive compensatory responses, preserving APLP2 function may help maintain the necessary, appropriately regulated compensatory mechanisms.

### 2.3 Early endosomes and protein trafficking

An endosome is a membrane-bound intracellular organelle that mediates the sorting, trafficking, and processing of internalized cargo following endocytosis. Early endosomes, in particular, play a central role in APP trafficking and processing (Figure 1), serving as key regulatory hubs that determine APP processing pathways and provide a platform for APP-dependent signaling. Through these functions, they regulate both the plasticity-related roles of APP and the production of A*β*. Studying these processes may help elucidate the pathological mechanisms of AD and lead to the discovery of novel therapeutic targets. However, therapeutic strategies that focus solely on reducing Aβ production may be ineffective. Within the framework of the maladaptive plasticity hypothesis, lowering Aβ levels without concomitantly altering levels of CTF-β may offer limited therapeutic benefit. Moreover, if a compound increases CTF-β levels, it could even accelerate disease progression. In FAD-linked presenilin mutations, *γ*-secretase-mediated cleavage of CTF-β is impaired, leading to altered cleavage sites and an increased Aβ42/40 ratio. Notably, certain presenilin mutations nearly abolish the generation of both Aβ40 and Aβ42 (Sun et al., 2017). On the other hand, across different FAD-linked presenilin variants, impaired γ-secretase function consistently leads to the accumulation of CTF-β (Kwart et al., 2019; Li et al., 2016). Although γ-secretase inhibitors have been extensively investigated as therapeutic candidates, reductions in Aβ production accompanied by increased CTF-β levels may exacerbate disease progression. Therefore, when designing drugs that target early endosomes and protein trafficking, it is crucial to assess the production of CTF-β, which contributes to the plasticity-related functions of APP.

Rab5 colocalizes with APP (Sabo et al., 2003) and is activated by CTF-β (Kim et al., 2016; Xu et al., 2016); thus, it may serve as a key regulator of APP-driven synaptic remodeling signaling (Figure 1). Overexpression of Rab5 (Pensalfini et al., 2020) and stabilization of its active form through overexpression of its adaptor protein, Adaptor Protein Containing PH Domain, PTB Domain, and Leucine Zipper Motif 1 (APPL1) (Jiang et al., 2025) induce tau phosphorylation. Conversely, in studies using Dp16 mice, antisense oligonucleotides targeting APP or Rab5 were reported to reduce tau hyperphosphorylation (Chen et al., 2025). Experimental findings indicate that overexpression of Ras and Rab Interactor 3 (RIN3), which interacts with the AD-related genes Bridging Integrator 1 (BIN1) and CD2-Associated Protein (CD2AP), may increase CTF-β levels and enhance tau phosphorylation through Rab5 activation (Shen et al., 2022). These findings suggest that Rab5 is involved in tau phosphorylation (Figure 1). Synaptic remodeling is tightly associated with cytoskeletal reorganization. Thus, elucidating the pathway by which Rab5 activation leads to tau phosphorylation may provide insights into the mechanisms by which APP signaling contributes to cytoskeletal reorganization during synaptic remodeling. Furthermore, understanding how dysregulation of this process results in tau hyperphosphorylation could lead to the identification of novel therapeutic targets. Nevertheless, because Rab5 is involved in essential cellular functions such as membrane trafficking and cell motility (Yuan and Song, 2020), the key challenge lies in preserving its physiological functions while selectively inhibiting its pathological signaling.

Sortilin-related receptor with A-type repeats (SORLA) is an endocytic protein-trafficking receptor that mediates the recycling of internalized APP back to the plasma membrane (Figure 1). Haploinsufficiency of the *Sortilin-Related Receptor 1* (SORL1) gene, which encodes SORLA, is causative of AD. AD-associated SORL1 mutations impair the ability of SORLA to sort APP away from early endosomes along the retromer-mediated recycling pathway. When APP recycling is impaired due to SORL1 deficiency, APP becomes excessively retained in early endosomes, leading to elevated CTF-*β* levels and enhanced APP-dependent synaptic remodeling. This dysregulation disrupts APP homeostasis and increases the risk of maladaptive synaptic plasticity. Mishra et al. reported that small-molecule enhancement of retromer activity can rescue SORLA functional deficits caused by AD-associated SORL1 mutations, restoring APP trafficking and normalizing early-endosomal handling (Mishra et al., 2023). These findings suggest that pharmacological correction of SORL1-related endosomal dysfunction could mitigate aberrant retention of APP in early endosomes and represent a promising therapeutic strategy. Pharmacological blockade of the PKCι/*λ*-β-arrestin2 axis upregulates SORLA levels, thereby reducing APP processing through the β-secretase pathway (Rehman et al., 2025). Similarly, 6-shogaol upregulates SORL1 expression, leading to decreased BACE1-mediated processing of APP and consequent reductions in both CTF-β and Aβ levels (Na et al., 2017). However, enhancing SORLA function may carry risks of unintended effects, given its broader role in membrane protein trafficking. Hence, therapeutic strategies targeting APP trafficking must balance efficacy with safety.

## 3 Targeting network hypersynchrony seen at early stage of the disease

### 3.1 Levetiracetam

Network hyperactivity has been observed prior to overt amyloid deposition (Koelewijn et al., 2019; Palmqvist et al., 2017; Quiroz et al., 2015; Su et al., 2015). One prevailing hypothesis suggests that soluble Aβ may drive this hyperexcitability. However, it remains hypothetical how soluble Aβ may induce such network hyperexcitability, despite its involvement in various neural processes (Palop and Mucke, 2016; Ray et al., 2024). FAD-linked mutations in APP and presenilin induce enhanced plasticity through increased production of CTF-β (Deyts et al., 2016; Kwart et al., 2019; Lee et al., 2010; Ray et al., 2024). In addition, as discussed in this paper, elevated Aβ production may serve as a signature of increased levels of its precursor CTF-β, thereby indicating that APP-induced enhancement of synaptic plasticity occurs in parallel with increased Aβ generation (Palmqvist et al., 2017). Thus, it is also plausible that APP-induced enhancement of synaptic plasticity may underlie the observed network hyperactivity (Kawabata, 2022a). This hypothesis is further supported by a logically coherent interpretation. Long-range neural network activity between distant brain regions is based on synchronized firing among principal neurons, coordinated by GABAergic interneurons within each region (Koelewijn et al., 2019). Direct activation of parvalbumin-positive GABAergic interneurons has been shown to enhance the power of gamma-frequency oscillations (Sohal et al., 2009). This finding suggests that enhanced synaptic plasticity may facilitate synchronized oscillatory activity through strengthened coupling between interneurons and principal neurons (Kawabata, 2022a). However, when coupling becomes excessive, glutamatergic input onto GABAergic interneurons may increase, potentially leading to their degeneration (Moga et al., 2002; Kawabata, 2022a). The loss of these interneurons not only promotes network hyposynchrony but also, conversely, gives rise to aberrant hypersynchronization when inhibitory control is compromised (Freund and Katona, 2007; Kawabata, 2022a). Separately, in older adults, task-dependent increases in neural activity have been observed (Grady, 2012), suggesting that hyperactive networks are functionally specific. Such selective activation may arise from enhanced synaptic plasticity leading to strengthened connectivity within specific neural circuits.

Therapeutic strategies aimed at counteracting network abnormalities have previously been proposed as strategies to improve cognitive functions (Palop and Mucke, 2016). For instance, suppressing the increased neural activity in the MTL observed during the transition from normal aging to MCI (Corriveau-Lecavalier et al., 2021b; Zott et al., 2018) may represent an effective therapeutic strategy for mitigating cognitive decline and improving prognosis. Levetiracetam, an antiseizure medication, modulates neurotransmitter release through binding to the synaptic vesicle glycoprotein 2A (SV2A). Low doses of levetiracetam (62.5 and 125 mg twice daily), but not the high dose (250 mg twice daily) have been shown to improve memory performance and reduce hippocampal hyperactivity in patients with MCI (Bakker et al., 2012, 2015). These findings support the notion that increased neural activity in the MTL reflects maladaptive connectivity associated with cognitive decline. In a small-scale randomized controlled trial involving 18 months of treatment with an extended-release formulation of levetiracetam (220 mg), no overall significant improvement was observed in the Clinical Dementia Rating Scale Sum of Boxes (CDR-SB). However, although not statistically significant, some numerical improvement was observed, especially in the ApoE ε4 noncarrier subgroup, where a 40% reduction in CDR-SB progression was reported (Mohs et al., 2024). Mohs et al. estimated that, when including both ApoE ε4 carriers and noncarriers in the overall population, a clinical trial with approximately 1,000 participants would be required to achieve sufficient statistical power to detect the difference in effect size observed in this trial. This mid-stage study may support undertaking a larger confirmatory trial. Maximizing its probability of success will require careful patient selection and sensitive, objective endpoints for assessing treatment effects.

### 3.2 Plasma phosphorylated-tau217

Plasma phosphorylated-tau217 (p-tau217) is an excellent biomarker to detect brain amyloid deposition. Moreover, it serves as a prognostic biomarker to predict future cognitive decline among cognitively unimpaired individuals (Ossenkoppele et al., 2025). Interestingly, plasma p-tau217 levels are significantly higher in newborns than in healthy individuals at any other age (Gonzalez-Ortiz et al., 2025). During development, phosphorylated tau is known to be present at high levels only during the period of intense neurite outgrowth (Brion et al., 1994), suggesting that elevated p-tau217 in newborns reflects intense synaptic plasticity. In cognitively unimpaired adults, elevations in p-tau217 may also reflect plastic neural changes. However, because higher p-tau217 levels in adults are associated with future cognitive decline, such increases are more likely to represent maladaptive rather than beneficial plasticity. Individuals carrying the ApoE ε4 allele exhibit network hyperactivity from ages long before the onset of overt cognitive impairment (Bookheimer et al., 2000; Dennis et al., 2010; Filippini et al., 2009). Previous study suggests that this phenomenon may arise because ApoE ε4, compared with other isoforms, more strongly enhances APP function, thereby predisposing neural circuits to excessive synaptic plasticity (Kawabata, 2022a). Among amyloid-negative cognitively healthy individuals, ApoE ε4 carriers show significantly higher p-tau217 levels than non-carriers (Martínez-Dubarbie et al., 2024). Although direct evidence demonstrating that increases in p-tau217 specifically reflect enhanced synaptic plasticity remains limited, the elevated p-tau217 levels observed in ApoE ε4 carriers may support the possibility that excessive plastic changes arise from very early, cognitively intact stages. These observations highlight the importance of treatment timing. In spontaneously epileptic rats, seizure-induced mossy fiber sprouting is suppressed only by prophylactic administration of levetiracetam (Sugata et al., 2011), indicating that treatment should begin as early as possible. Similarly, the limited therapeutic benefit observed in ApoE ε4 carriers in a clinical trial of extended-release levetiracetam (Mohs et al., 2024) may reflect suboptimal timing of administration. When the therapeutic goal is to suppress age-related neural hyperactivity, as in the case of levetiracetam, incorporating an appropriate range of plasma p-tau217 concentrations into the inclusion criteria may enable optimal patient selection for intervention. In addition, low doses of levetiracetam have been shown to alleviate kainic acid-induced memory deficits and reduce aberrant tau phosphorylation in APP23/MAPT mice (Zheng et al., 2022). It would be of great interest to determine whether p-tau217 serves as a surrogate biomarker for evaluating the clinical efficacy of drugs like levetiracetam.

Levetiracetam was shown to modulate APP metabolism by favoring the non-amyloidogenic *α*-pathway over the amyloidogenic β-pathway, thereby reducing levels of CTF-β and Aβ (Rao and Savas, 2021). APP and BACE1 shuttle between the plasma membrane and early endosomes via the endocytic pathway. Importantly, early endosomes are also engaged in synaptic vesicle recycling, serving as hubs for the sorting and recycling of membrane proteins. Levetiracetam binds to the synaptic vesicle protein SV2A and modulates synaptic vesicle cycling. Therefore, it is conceivable that levetiracetam, possibly via modulation of early endosomal dynamics, indirectly reduces the frequency of APP internalization and its encounters with BACE1 in early endosomes, thereby resulting in a relative predominance of the non-amyloidogenic pathway. According to the maladaptive plasticity hypothesis, levetiracetam may suppress excessive plastic responses by reducing CTF-β-mediated APP signaling, thereby alleviating neuronal network hyperexcitability. Notably, levetiracetam has not been associated with prominent adverse effects on cognitive function (Bakker et al., 2012, 2015; Mohs et al., 2024). By targeting SV2A, it may preferentially dampen exuberant synaptic remodeling while sparing adaptive forms of plasticity, thus avoiding overt cognitive side effects. As a potential mechanism of action, it can be hypothesized that the modulatory effect of levetiracetam on APP processing becomes more pronounced under conditions in which APP pathologically accumulates within early endosomes.

### 3.3 Microglia

Microglia critically regulate synaptic plasticity (Andoh and Koyama, 2021). Early network hyperexcitability induced by the AD-risk Triggering Receptor Expressed on Myeloid Cells 2 (TREM2) R47H variant has been demonstrated in knock-in mice (Das et al., 2023), whereas age-dependent synaptic degeneration has been observed in the non-cryptic splice site (NSS) R47H knock-in model that corrects the splicing artifact present in earlier lines (Tran et al., 2023). Early network hyperexcitability is thought to result from impaired synaptic pruning caused by reduced TREM2 function (Das et al., 2023). It is noteworthy that reduced TREM2 function is associated with pathological changes resembling those observed in humans and mouse models carrying FAD-linked mutations, namely an early phase of network hyperexcitability followed by a subsequent decline in network activity. From this perspective, within the framework of the maladaptive plasticity hypothesis, TREM2 agonists may represent potential therapeutic candidates for AD. However, the recent INVOKE-2 clinical trial failed to demonstrate significant clinical benefits of the TREM2-targeted agonist antibody AL002 (Ma et al., 2025). Given that synaptic plasticity is regulated in a highly complex manner across multiple stages, formation, maturation, and pruning, it is possible that TREM2 agonists may exert therapeutic effects only in individuals harboring AD-risk TREM2 variants, rather than in the broader AD population.

### 3.4 Other target molecules and their evaluation systems

AD mouse models expressing FAD-linked mutant genes exhibit a wide range of network abnormalities. Previous study has proposed that such network dysfunction may arise from abnormalities in synaptic plasticity caused by mutations in APP or presenilin genes (Kawabata, 2022a). It has also suggested that the diverse phenotypes observed across models may reflect differences in the patterns and expression levels of the transgenes, as well as complex neural responses triggered by network hyperexcitability-induced neurodegeneration (Kawabata, 2022a). Conditional deletion of BACE1 in adulthood has been shown to ameliorate epileptiform activity in 5 × FAD mice (Yao et al., 2023). Although the phenotypic diversity of AD mouse models complicates interpretation, the observation that genetic deletion of BACE1, already considered a therapeutic target, can rescue aberrant network activity suggests that network-level readouts in these models may provide a useful platform for compound screening and target identification in drug discovery.

One example is riluzole, a drug approved for amyotrophic lateral sclerosis, which reduces excessive excitability mediated by glutamatergic transmission. In APP/PS1 mice, administration of riluzole during the 2-6-month period, when hippocampal hyperglutamatergic signaling is evident, resulted in procognitive effects and a reduction in glutamatergic tone, with effects persisting for 6 months after treatment discontinuation (Hascup et al., 2021). In a pilot clinical trial in patients with AD, 6 months of riluzole treatment attenuated the decline of glucose metabolism in several prespecified regions, including the PCC/precuneus, although cognitive benefits have not yet been demonstrated (Matthews et al., 2021). Another example involves galectin-3 (Gal3). In 5 × FAD mice lacking Gal3, gamma-band oscillatory activity was preserved at levels similar to those of wild-type mice (Arroyo-García et al., 2023). Moreover, Aβ plaque burden was reduced (Arroyo-García et al., 2023; Boza-Serrano et al., 2019), and cognitive performance was improved (Boza-Serrano et al., 2019). These findings indicate that Gal3 plays a significant role in the emergence of AD-related network dysfunction and amyloid pathology. Nevertheless, whether Gal3 inhibition represents a viable therapeutic strategy remains unclear, as its broader physiological functions are still being elucidated.

## 4 Activity-based interventions and drug combinations

### 4.1 Synaptic plasticity and activity-based interventions

Spinal cord injury (SCI) induces changes in the spinal cord that produce severe impairment of motor, sensory, and autonomic functions. Following SCI, spontaneous synaptic remodeling occurs both within the spinal cord and in supraspinal structures. While some of these changes support recovery, others are maladaptive and may hinder functional restoration, for example, in the development of neuropathic pain, a common secondary complication of SCI. Activity-based interventions (ABIs), such as exercise training (ET), which promote activity-dependent synaptic remodeling, can enhance sensorimotor recovery not only by fostering adaptive plasticity but also by mitigating maladaptive synaptic connectivity (Calderone et al., 2024; Chen et al., 2022; Lynskey et al., 2008; Walker and Detloff, 2021). In SCI, spontaneous synaptic remodeling, which may result in maladaptive connections, occurs within days to weeks post-injury (Lynskey et al., 2008), during a period of intense plastic changes aimed at repairing the damaged nervous system (Wu et al., 2025). At-level neuropathic pain, which is localized around the site of injury and caused by damage to nerves at that level, tends to emerge more frequently during the acute phase following SCI (Burke et al., 2017). Accordingly, initiating ABIs early in the recovery process may help steer this heightened plasticity toward adaptive outcomes, thereby mitigating maladaptive changes and enhancing functional recovery. Neuronal plasticity following stroke also emerges within hours to days and continues for weeks. Within 1–3 months post-stroke, intervention-mediated recovery is often most pronounced (Hara, 2015). Similar to SCI, spontaneous plasticity during this period may contribute to maladaptive reorganization; however, appropriately timed rehabilitation can promote adaptive plasticity, potentially supporting functional recovery (Hara, 2015; Nudo, 2006). After stroke, if plastic changes progress excessively or aberrantly, they can also result in neuronal hyperexcitability, leading to late seizures that typically emerge 1 week or more after stroke onset and to excitotoxic neuronal damage (Adhikari et al., 2023; Altman et al., 2019). Collectively, although spontaneous plasticity emerges rapidly after injury, it is often diffuse and non-selective, and may not reliably support functional restoration; rather, it may contribute to functional impairment and to neuronal hyperexcitability leading to excitotoxicity. In contrast, activity-dependent plasticity can induce targeted synaptic modifications that align with functional goals, thereby fostering adaptive outcomes (Dunlop, 2008).

### 4.2 Implications for Alzheimer’s disease

ET has been shown to produce cognitive benefits in older adults (Chirles et al., 2017; Nishiguchi et al., 2015; Smith et al., 2013; Voss et al., 2010; Won et al., 2021). Although there is no direct evidence for dementia prevention, ET is considered one of the most promising strategies for preventing dementia. In a 12-week ET program, improvements in cognitive function were accompanied by increased connectivity within the DMN in the PCC/precuneus and MTL of patients with MCI (Chirles et al., 2017; Won et al., 2021). Of note, ET also improves memory performance while reducing task-related neural activation in cognition-associated regions. A 1-year ET (walking) program not only improved functional connectivity within the DMN, which is reduced with aging, but also led to a decrease in connectivity between the frontal executive network and DMN, regions that show greater baseline connectivity in older adults than in young adults (Voss et al., 2010). In a randomized controlled trial of a 12-week physical and cognitive exercise program for community-dwelling older adults, participants in the program-implementation group showed greater post-intervention improvement in memory and executive function. In addition, after the intervention, less activation was found in brain regions associated with short-term memory in the program-implementation group (Nishiguchi et al., 2015). Similarly, in MCI patients, a 12-week ET program improved semantic memory performance and decreased brain activation related to semantic memory retrieval (Smith et al., 2013).

**Figure 2:**
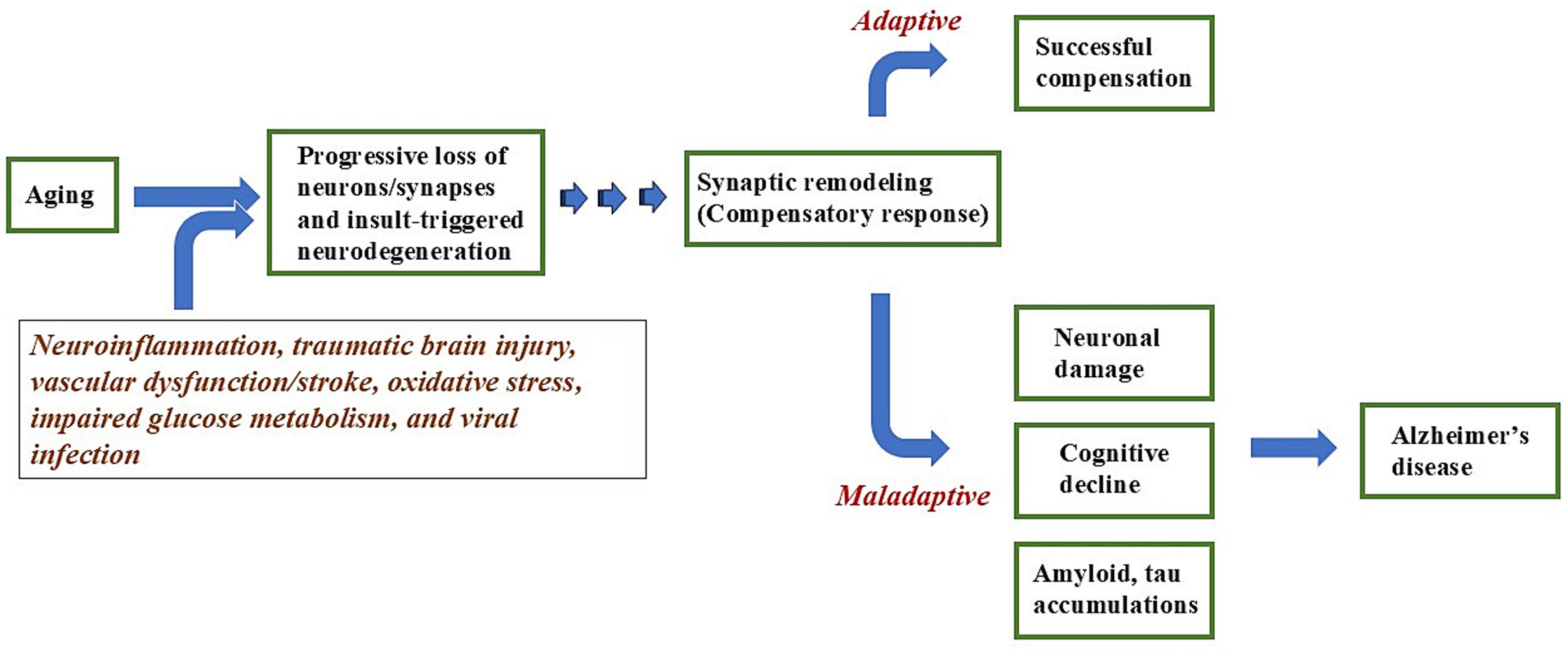
Proposed mechanism of how Alzheimer’s disease is developed with aging. Aging, neuroinflammation, traumatic brain injury, vascular dysfunction, oxidative stress, metabolic impairment, and viral infection trigger synaptic remodeling. While initially compensatory, dysregulated remodeling becomes maladaptive, leading to synaptic dysfunction, neuronal vulnerability, and the emergence of Alzheimer’s disease-related pathology.

**Figure 3:**
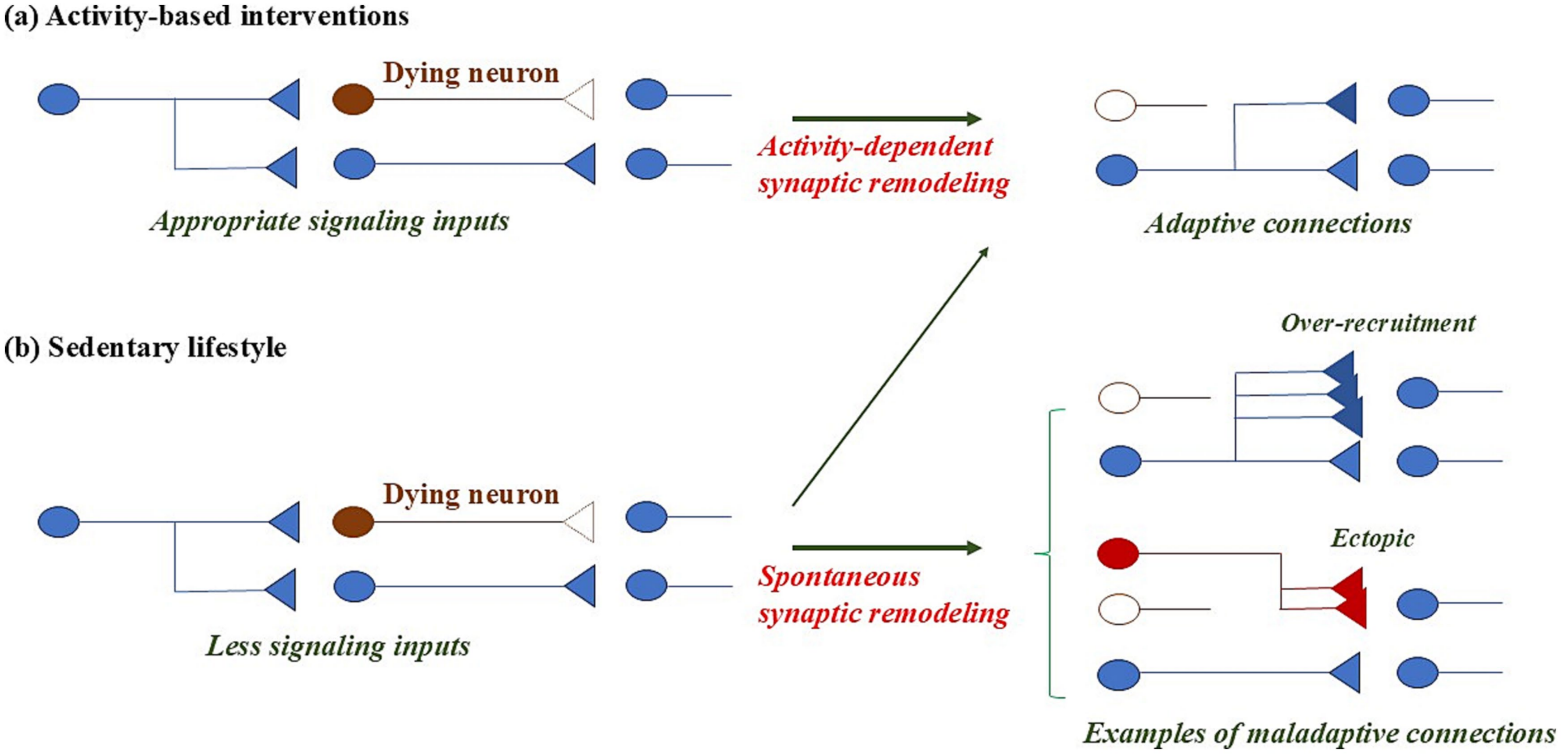
Compensatory responses driven by activity-dependent and spontaneous synaptic remodeling. Activity-dependent interventions promote adaptive remodeling that strengthens appropriate circuit connectivity **(a)**. By contrast, low-activity states promote spontaneous, unregulated remodeling, increasing the risk of maladaptive synaptic changes that destabilize network function **(b)**.

AD is a neurodegenerative disorder that disrupts neuronal networks responsible for higher cognitive functions. Synaptic plasticity plays a pivotal role in cognition and occurs routinely in response to learning, information acquisition, environmental stimuli, and training (Citri and Malenka, 2008). With advancing age, the progressive loss of neurons and synapses may impose an increasing demand for compensatory remodeling within neural networks that routinely undergo plastic changes (Burke and Barnes, 2006; Grady, 2012; Petralia et al., 2014). Unlike in stroke or SCI, where plasticity is temporally restricted, remodeling demands in older adults may persist throughout life and become increasingly complex. Consequently, the risk of spontaneous synaptic remodeling and the resulting maladaptive connectivity may persist continuously (Figure 2). During the transition from normal aging to MCI, compensatory responses increasingly occur, some of which may represent maladaptive neural connectivity associated with cognitive decline and the onset of AD (Corriveau-Lecavalier et al., 2021a; Yassa et al., 2010; Dickerson et al., 2004; Dickerson and Sperling, 2008; Huijbers et al., 2015; Miller et al., 2008; O'Brien et al., 2010). Although the mechanisms underlying the cognitive benefits of ET may not necessarily be attributable to plasticity (Tari et al., 2025), during the transition from normal aging to MCI, a period characterized by heightened demands for plasticity, one possible hypothesis is that ET promotes adaptive plasticity while mitigating maladaptive synaptic connectivity, analogous to the effects of ABIs in stroke or SCI recovery. When ABIs are applied to older adults, activity-dependent synaptic remodeling is appropriately directed, allowing compensatory responses to occur in a regulated manner and restoring balanced brain activity (Figure 3a). In contrast, a sedentary lifestyle may lead to unregulated spontaneous synaptic remodeling, increasing the likelihood of unsuccessful compensation and resulting in maladaptive synaptic connections (Figure 3b). At the whole-brain level, this period is characterized by the coexistence of hypoactive and hyperactive networks (Bai et al., 2008; Qi et al., 2010; Talwar et al., 2021; Wang et al., 2018). ABIs may enhance activity in networks that decline with aging, while simultaneously preventing further exacerbation of hyperactive networks observed in older adults, potentially even contributing to their normalization. Thus, ABIs may represent an ideal approach for the prevention of cognitive decline during the transition from normal aging to MCI within the framework of the maladaptive plasticity hypothesis.

### 4.3 Combining with pharmacological treatments

A clinical trial has been launched to evaluate the combined therapy of multidomain lifestyle interventions, consisting of diet, exercise, cognitive training, cardiovascular and metabolic risk management, and social engagement, together with the antidiabetic drug metformin. This endeavor is expected to serve as a foundation for future novel combinations of ABIs and pharmacological treatments (Barbera et al., 2023), highlighting the potential of developing combination therapies that incorporate ABIs with drugs such as levetiracetam. Implementation in society will require appropriate policy design. Pharmacological agents should be approved specifically for rehabilitation-eligible individuals and covered under the applicable health insurance framework. In parallel, whether the provision of ABIs to patients receiving such pharmacological agents will be recognized as reimbursable rehabilitation services, the definition and coverage of which vary across countries, remains to be discussed, and modifications to reimbursement system design are likely to be required for their implementation. Accumulating evidence is expected to provide further guidance for this debate. In a cross-sectional study of 1,144 participants with a mean age of 70.9 years, individuals with higher levels of physical activity had significantly lower p-tau217 compared with those with lower levels of activity. Moreover, greater physical activity was associated with higher MMSE scores, lower CDR-SB scores, and overall, better preserved cognitive function (Kim et al., 2025). Given the cross-sectional design of this study, causal relationships cannot be established at present. To determine whether ABIs can reduce p-tau217 levels in parallel with cognitive improvements, future randomized controlled trials using exercise interventions are warranted. Should ABIs be shown to suppress p-tau217, this biomarker could be utilized as a surrogate marker for ABIs. In clinical trials of ABIs, blinding is inherently difficult; therefore, having such an objective marker would help generate more robust evidence.

### 4.4 Neuromodulation

Neuromodulation refers to interventions that externally alter or regulate neural activity using physical or biological stimuli, including light, sound, sensory stimulation, ultrasound, electrical or magnetic stimulation, vagus nerve stimulation, spinal stimulation, optogenetics, and deep brain stimulation. Among these approaches, 40-Hz sensory stimulation has gained attention as a potential therapeutic strategy for AD (Adaikkan and Tsai, 2020; Iaccarino et al., 2016). Early clinical studies demonstrated that 40-Hz Non-invasive Gamma ENtrainment Using Sensory stimulation (GENUS) can induce gamma entrainment in both cortical and subcortical regions and that 3 months of daily 40-Hz light-and-sound stimulation is associated with reduced ventricular enlargement, attenuated hippocampal atrophy, enhanced functional connectivity, improved associative memory, and more stable activity rhythms (Chan et al., 2022). There is ongoing debate regarding whether 40-Hz sensory stimulation can influence deep brain structures. While 40-Hz flicker stimulation alone does not reliably induce intrinsic gamma oscillations in deep regions such as the hippocampus (Soula et al., 2023), other studies have reported that performing cognitive tasks concurrently with 40-Hz visual stimulation enhances the strength and spatial extent of gamma entrainment, promoting its propagation to additional neural areas, including deep structures that are not recruited during passive stimulation (Khachatryan et al., 2022). Neuromodulation can augment the effects of rehabilitation; for example, in patients with Parkinson’s disease, rhythmic auditory stimulation enhances rehabilitation outcomes and motor function (Wang et al., 2022b). ABIs may help redirect maladaptive plasticity toward more adaptive patterns and could potentially increase responsiveness to 40-Hz sensory stimulation, although the evidence is limited and context-dependent. Taken together, these observations suggest that combining ABIs with neuromodulation approaches, such as 40-Hz light-and-sound stimulation, may yield synergistic benefits, with possible relevance to cognitive function.

## 5 Discussion

The target Aβ species of lecanemab (van Dyck et al., 2023) and donanemab (Sims et al., 2023) are soluble Aβ protofibrils and aggregated Aβ in amyloid plaques, respectively. A common observation with both agents is the substantial reduction of amyloid burden in the brain. However, in lecanemab, no correlation was observed between changes in amyloid PET centiloid values and changes in CDR-SB following treatment. Pearson correlation coefficients at 6, 12, and 18 months (ranging from −0.016 to −0.165 in the active group and −0.053 to −0.117 in the placebo group) were close to zero, indicating a lack of meaningful association between amyloid reduction and clinical improvement.^2^ Similarly, in donanemab, reductions in amyloid PET showed no significant correlation with changes in the Integrated Alzheimer’s Disease Rating Scale or CDR-SB up to 76 weeks.^3^ Within the Aβ hypothesis, it remains controversial which Aβ species are responsible for disease progression and which mechanisms of action are clinically relevant. Given that lecanemab and donanemab demonstrate clear yet limited clinical benefits, other causal factors should also be considered. Drugs targeting tau are under active investigation, and the outcomes of ongoing trials are awaited with interest (Harris et al., 2025). Within the amyloid hypothesis, early amyloid deposition is considered capable of initiating the AD cascade; however, Aβ alone is viewed as insufficient to drive disease progression, and tau pathology is recognized as a critical determinant of neurodegeneration and cognitive decline.

Beyond the amyloid hypothesis, several other pathological processes, including neuroinflammation, traumatic brain injury, vascular dysfunction/stroke, oxidative stress, impaired glucose metabolism, and viral infection have been extensively studied as major areas of investigation in AD research. While each of these mechanisms can induce neurodegeneration, the question of why they ultimately converge on the characteristic pathological hallmarks of AD, namely APs and NFTs, remains unresolved. An important point is that both APs and NFTs may represent downstream manifestations of excessive neuronal plasticity (Kawabata, 2022a). Following cerebral ischemia, BACE1 upregulation, enhanced Aβ production, and tau hyperphosphorylation have been reported (Basak et al., 2023; Chen and Jiang, 2019; Nguyen et al., 2018). Since stroke triggers intense neuronal remodeling and synaptic reorganization (Adhikari et al., 2023; Altman et al., 2019; Hara, 2015; Nudo, 2006), it is conceivable that this surge of plastic activity may accelerate AD pathology and increase the risk of the development of AD (Saiz-Vázquez et al., 2025). Similarly, emerging evidence suggests that Severe Acute Respiratory Syndrome Coronavirus 2 (SARS-CoV-2) infection may not only increase the risk of subsequent AD (Wang et al., 2022a), but also exacerbate AD-related pathological processes (Duff et al., 2025). Functional neuroimaging studies have demonstrated maladaptive forms of plasticity after Corona Virus Disease 2019 (COVID-19) infection, characterized by hypersynchrony of neuronal networks, which is associated with post-COVID cognitive dysfunction (Carreras-Vidal et al., 2025; Dacosta-Aguayo et al., 2024). Collectively, these findings suggest that compensatory mechanisms triggered by ischemic, metabolic, or viral insults may, when they become maladaptive, increase the risk of AD onset and act as a common pathway in AD pathogenesis. Neurodegeneration arising from diverse etiologies may, through the progressively increasing demand for compensatory synaptic adaptations with aging, raise the likelihood of maladaptive synaptic remodeling, thereby accelerating the trajectory toward the onset of AD (Figure 2). Traditional models of AD pathogenesis, including the amyloid hypothesis, have undergone increasing re-evaluation in recent years, and multiple new theoretical frameworks have been proposed. Recent reviews emphasize that AD is a multifactorial disorder involving parallel disruptions across multiple biological systems (Gyimesi et al., 2024; Rajendran and Krishnan, 2024), prompting alternative frameworks such as models that link metabolic stress to downstream amyloid and tau pathology (Mackey-Alfonso and Barrientos, 2025). Within this broader context, the maladaptive plasticity hypothesis provides an integrative perspective by proposing that diverse upstream insults converge on aberrant synaptic plasticity, which becomes maladaptive and drives network dysfunction and clinical progression.

In this paper, we have discussed preventive and therapeutic strategies based on the maladaptive plasticity hypothesis, which posits that the accumulation of amyloid and tau arises as a consequence of excessive synaptic remodeling. When plasticity is abnormally enhanced, it not only triggers such accumulations but also disrupts cytoskeletal homeostasis and promotes neurodegeneration. At the same time, it fosters maladaptive neuronal connections that lead to cognitive impairment and neuronal death caused by glutamate excitotoxicity and energy failure. Therefore, targeting maladaptive plasticity may lead to substantial clinical benefits. Importantly, this conceptual framework is not limited to AD but can also be applied to therapeutic strategies for traumatic brain injury, stroke, and viral infection, conditions in which excessive or dysregulated plasticity similarly may contribute to poor outcomes. Specifically, administering agents that suppress maladaptive plasticity, such as levetiracetam, during the acute phase after the insult may prevent spontaneous, aberrant plastic changes that worsen long-term outcomes, thereby improving recovery and potentially reducing the subsequent risk of developing AD. Such an approach is likely to be particularly effective when combined with ABIs. In the case of COVID-19, individuals at elevated risk for post-infection cognitive decline have already been identified, including older adults, patients with severe disease, those who experienced delirium, and individuals with underlying conditions such as hypertension, chronic obstructive pulmonary disease (COPD), or cardiovascular disease (Liu et al., 2021). Targeted pharmacological intervention limited to these high-risk groups may therefore represent a feasible and rational strategy.

Neuronal plasticity is indispensable for brain function, and indiscriminate suppression would inevitably impair cognition and behavior. Therefore, therapeutic strategies should aim to selectively constrain maladaptive forms of plasticity while preserving adaptive ones. ABIs may achieve this balance by suppressing spontaneous maladaptive remodeling and enhancing adaptive processes. From a pharmacological perspective, SV2A modulation represents a promising approach for attenuating maladaptive hyperexcitability, and suppression of APP expression may likewise mitigate pathology with minimal adverse effects. Continued research into the functional aspects of APP will help advance the development of novel therapeutics capable of maintaining essential plasticity while preventing its pathological dysregulation.

## Data Availability

The original contributions presented in the study are included in the article/supplementary material, further inquiries can be directed to the corresponding author.

## Footnotes

1
https://www.alzforum.org/therapeutics/mivelsiran

2
https://www.pmda.go.jp/drugs/2023/P20230919001/170033000_30500AMX00272_A101_1.pdf

3
https://www.pmda.go.jp/drugs/2024/P20240920001/530471000_30600AMX00243000_A100_1.pdf
